# Acute Correction of Femoral Malrotation After Intramedullary Nailing Using an Intraoperative Digital Protractor

**DOI:** 10.7759/cureus.37108

**Published:** 2023-04-04

**Authors:** Joshua Goethals, Daniel R Cavazos, Marek Denisiuk, Ryan Bray, Kerellos Nasr, Rahul Vaidya

**Affiliations:** 1 Orthopaedic Surgery, Detroit Medical Center, Detroit, USA; 2 Orthopaedic Surgery, Wayne State University School of Medicine, Detroit, USA

**Keywords:** low dose ct, ct scanogram, iatrogenic lld, prospect trial, second generation nail, inherent anteversion, retroversion, femoral malrotation, femoral anteversion, intramedullary nailing

## Abstract

Objective: The goal of the study is to diagnose and accurately correct malrotation of femur fractures after intramedullary (IM) nailing.

Materials and methods: An institutional review board (IRB) approved prospective study that was performed at a U.S. level 1 trauma center. After IM nailing of comminuted femur fractures, a computed tomography (CT) scanogram was routinely performed to detect the difference in the postoperative femoral version. Patients with malalignment greater than 15 degrees compared to the contralateral side were informed about the discrepancy and offered to have it acutely corrected. A four-pin technique was used: two Schanz pins were used for measuring angles and two different pins were used to turn and correct the malalignment. The pin in the distal fragment is placed directly under the nail to prevent shortening in comminuted fractures. The nail was unlocked either proximally for retrograde nails or distally for antegrade nails. The Bonesetter Angle application was used as a digital protractor to intraoperatively measure the two reference pins and correct the malrotation. Alternate holes were used for relocking the nail. All patients received a CT scanogram after correction.

Results: 19/128 patients with comminuted femoral fractures over five years with malrotations between 18 and 47 degrees were included in the study with an average malrotation of 24.7 + 8 degrees. All patients were corrected to an average of 4.0 +/- 2.1 degrees difference, as compared to the contralateral side (range 0-8). No patients required further surgeries to correct malrotation.

Conclusion: Comminuted fractures with malrotation >15 degrees after femoral nailing have an incidence of 15% at our institution. This technique provides an efficient and accurate correction method with the use of an intraoperative digital protractor, avoiding the need for revision IM nailing or osteotomies.

## Introduction

Fractures of the femoral shaft are one of the most common injuries treated by orthopedic surgeons with an incidence of 10-21 per 100,000 per year [1]. These fractures are typically the result of high-energy trauma and are often associated with significant bony comminution. The gold standard of treatment for these injuries is locked intramedullary (IM) nailing which has resulted in high union rates. One of the most common complications of locked IM nailing is femoral malrotation with an incidence of 19%-56% [2-14], which is especially prevalent in highly comminuted fractures and transverse fractures due to a lack of osseous integration at the fracture site to assist in anatomic alignment [2-9]. Malrotation of greater than 15 degrees results in functional complaints such as anterior knee pain with demanding activities such as running, climbing stairs, and sports. Internal rotation is better tolerated than external rotation [4,5,7].

Several techniques have been proposed to avoid femoral malrotation intraoperatively; unfortunately, femoral malrotation during IM nailing still occurs [11-17]. To the best of our knowledge, no technique has been described to correct malrotation early during the initial hospitalization and before bony healing occurred [18].

The purpose of this study is to 1) demonstrate a technique to correct the malrotation after locked femoral nailing, 2) present our initial case series, and 3) evaluate its accuracy.

## Materials and methods

This is a prospective institutional review board (IRB) approved study (020717MP4E) that was performed over a five-year period between 2016 and 2021. One hundred twenty-eight patients with Winquist III and IV femur fractures underwent locked IM nailing followed by a low-dose computed tomography (CT) scanogram in the immediate (24hrs) postoperative period. The protocol utilizes CT technology to administer 10% of the ionizing radiation of a standard CT scan of the lower extremity to determine bilateral leg lengths and femoral version [11]. Of our cohort, 19 of those patients were found to have a rotational malalignment >15 degrees compared to their contralateral femur using this low-dose CT protocol.

**Figure 1:**
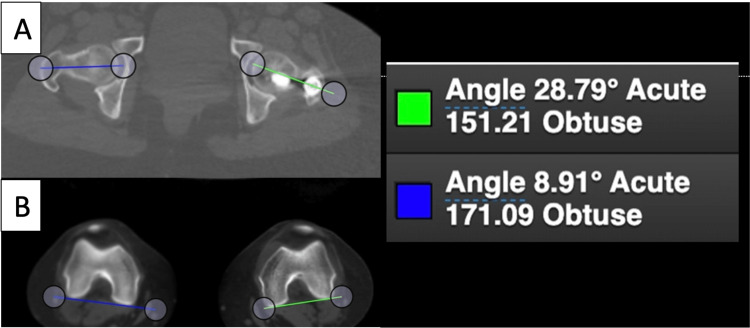
Measurement of femoral version based on CT scanogram axial views. (A) Femoral neck angles bilaterally. (B) Measurement of the posterior condylar axis bilaterally. Legend on right showing the right femur (blue lines) to be anteverted 8.91 degrees and the left femur (green lines) to be anteverted 28.79 degrees. ° = degree

Femoral version measurements were conducted using the Bonesetter application (Detroit, MI) with axial cuts from CT scanograms (Figures 1A, 1B) [17]. Proximal angle measurements were taken in alignment with the femoral neck, while distal angle measurements were taken in alignment with the posterior side of the femoral condyles. Each measurement was validated by three observers for the most appropriate positioning of the aforementioned lines. Femur, tibia, and overall leg lengths were measured from the CT scout image as part of the postoperative protocol for comminuted fractures at our institution. Limb length was assessed by selecting the superior most point on the femoral head to the center of the plafond. Femur length was assessed by measuring from the superior most point on the femur to the most distal aspect of the medial femoral condyle. Tibia length was assessed by measuring from the intercondylar eminence to the center of the plafond [17]. Outcome measurements included the version of the contralateral and operative femur and the angle of each proximal locking screw in comparison to the centerline of the femoral neck.

Technical procedure

The technique is performed utilizing 4 x 5.0mm Schanz pins strategically placed throughout the affected limb. One pin is used to stabilize length, one pin to hold the proximal fragment which are used to perform the derotation, and two pins are used to reference the correction.

**Figure 2:**
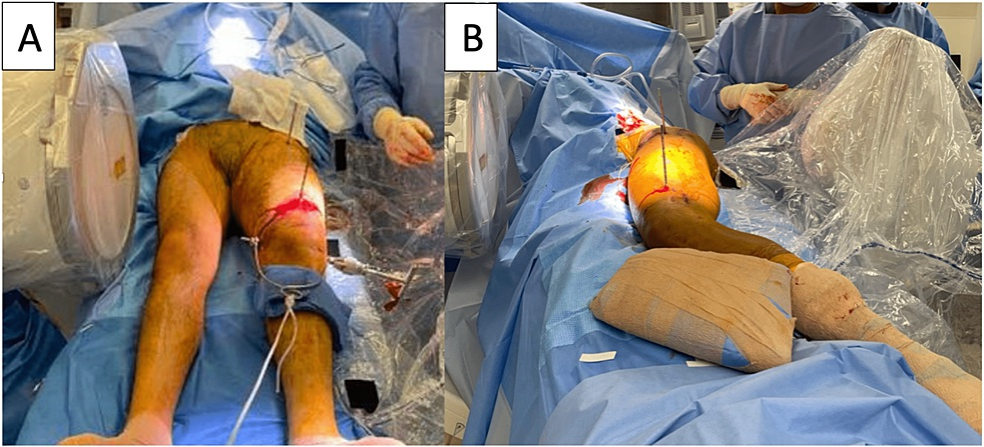
Intraoperative image demonstrating patients undergoing a femoral derotation procedure for femoral malrotation. It can be done supine (A) or lateral (B). One or both extremities may be sterilely prepped depending on the surgical plan.

**Figure 3:**
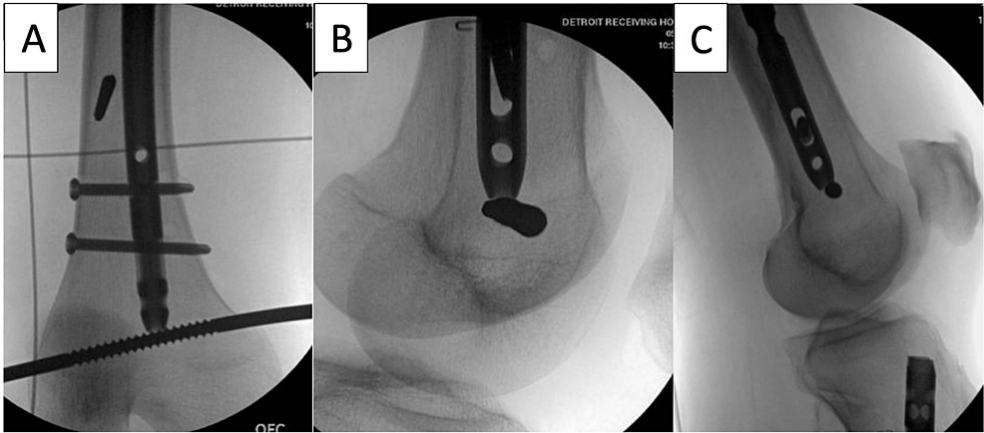
In the case of comminution and a concern for loss of leg length while doing this procedure, a 5.0 mm Schanz pin can be placed perpendicular to the nail abutting the distal tip of the nail as seen on the anterior-posterior (AP) radiograph (A), oblique (B) and lateral (C). This prevents shortening at the femoral fracture site when the distal locking screw is removed. This is a maneuvering pin and not reference pin.

**Figure 4:**
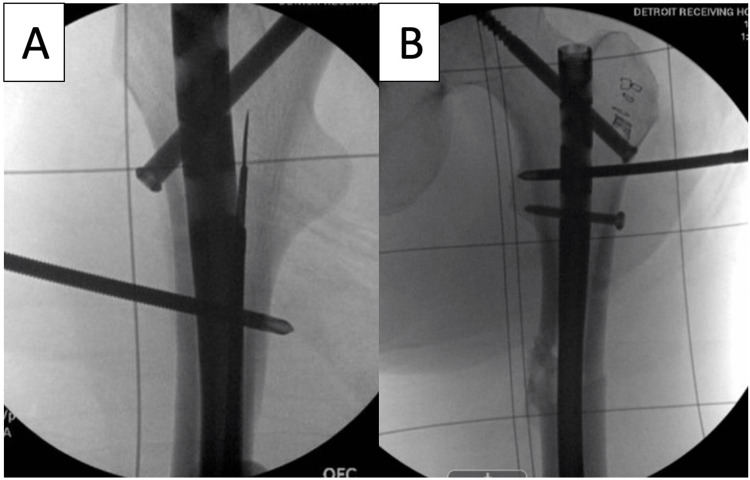
A 5.0 mm Schanz pin is placed into the femur proximal to the fracture site which can be placed in the shaft around the nail (A) or in the trochanteric region (B). This pin is used as a post to hold the proximal fragment in place so that the distal fragment can be rotated.

**Figure 5:**
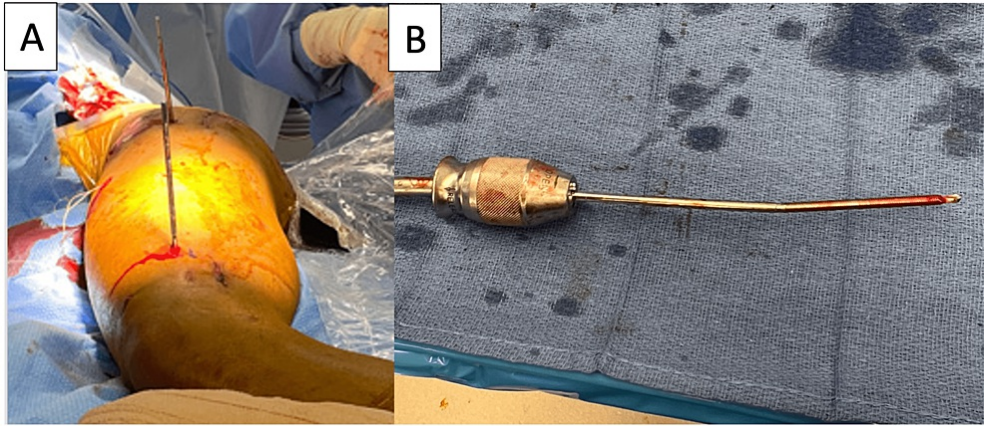
In the lateral position, two Schanz pins are placed in the femur. They must be placed in parallel in the same plane (A). These pins will be used as reference pins and are not used for manipulation of the extremity. The manipulating pins almost always bend and using them will distort the correction (B).

Under fluoroscopic guidance, a 5.0 mm Schanz pin is placed from lateral to medial immediately adjacent to the distal aspect of the nail (Figures 2A, 2B). This is immediately distal to an antegrade nail or immediately proximal to a retrograde nail. This step is particularly important as it serves to prevent shortening of the fracture site over the nail after the distal locking screws are removed, especially in length-unstable fractures (Figures 3A-3C). Another 5.0 mm Schanz pin is placed into the proximal femoral segment, perpendicular to the femoral shaft and proximal to the fracture site. This pin may be placed into the shaft around the nail if space allows or in the trochanteric region (Figures 4A, 4B) in antegrade nails and distally around the nail in retrograde nails. The proximal pin acts as a post while the distal pin is used to rotate the distal fragment to obtain rotational correction. Two reference pins are placed: the first into the proximal segment and the second into the distal femoral segment. They are placed in a parallel configuration to each other (Figures 5A, 5B). These two pins are then used to reference the correction created by the derotational maneuver. These pins are not to be manipulated as any force on the pins can induce a bend or loosen at the pin-bone interface resulting in inaccurate correction measurement.

**Figure 6:**
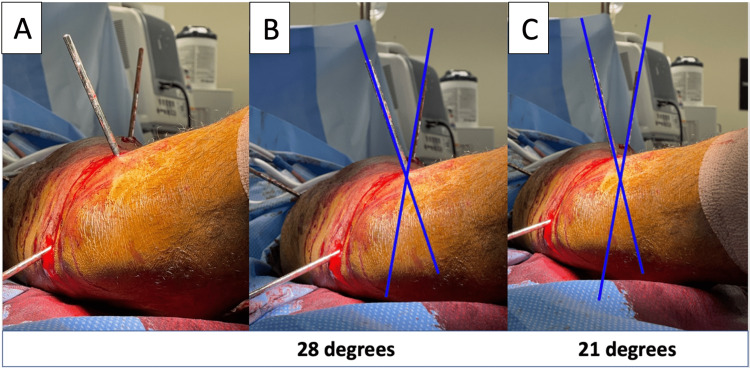
Intraoperative imaging demonstrating the derotational maneuver. The rotation is performed by two separate pins and adjusted and measured until the right angle is achieved (A). Intraoperatively, the application (Bonesetter Angle App) image demonstrating the measured correction angle (degrees) obtained from the derotational maneuver before (B) and after (C). Blue lines overly the two reference pins.

**Figure 7:**
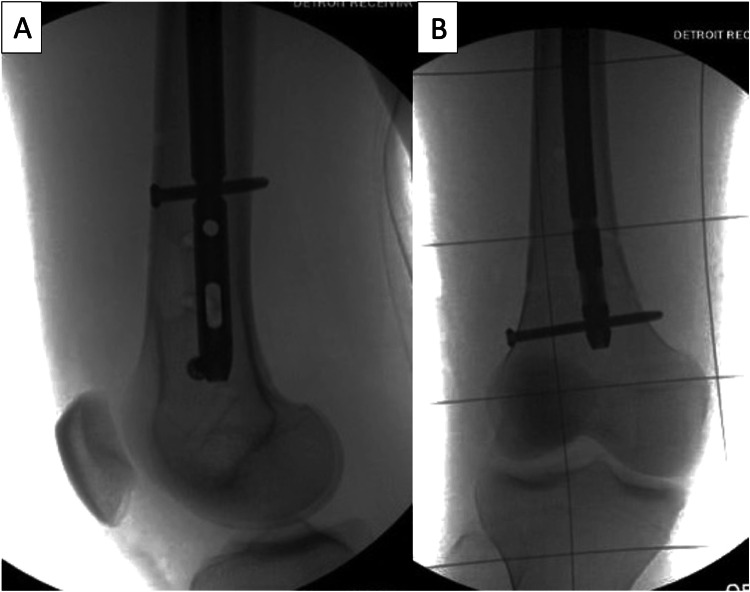
Distal interlocking screws are placed in the previously unused interlocking holes. Once the distal interlocking screw(s) is placed, the rotation is locked in place and all pins can be removed as seen on the lateral (A) and AP (B) radiographs.

**Figure 8:**
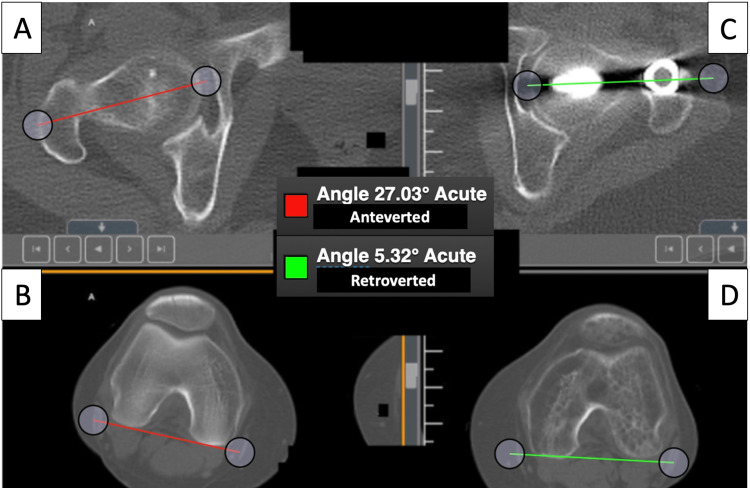
Pre-correction CT scanogram with pre-correction femoral rotation measurement obtained for the left and right extremity with the Bonesetter application. The contralateral femur (A, B) measures 27 degrees of anteversion (red lines). The operative femur (C, D) with the presence of an IM nail measured 5 degrees of retroversion (green lines). A 32-degree difference is outside the acceptable range of <15 degrees compared to the contralateral side. ° = degree

**Figure 9:**
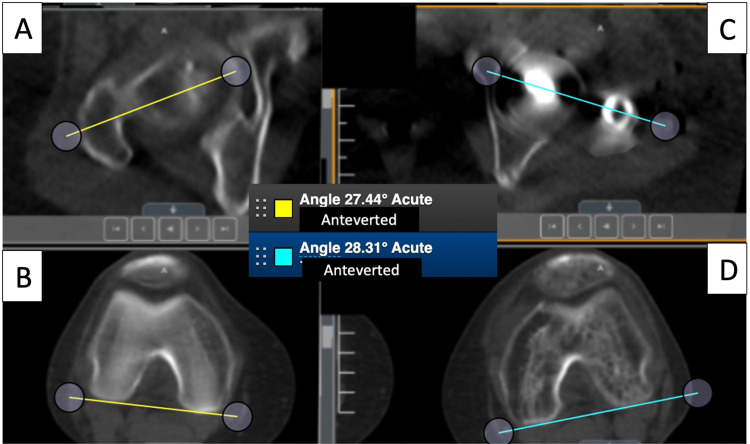
Post-correction CT scanogram of both femurs. Right femoral anteversion (A, B) is unchanged 27 degrees (yellow). The left femur (C, D) is measured to be in 28 degrees of femoral anteversion (light blue lines). There is a 0.75 deg difference between the two femurs which is in the acceptable range of <15 deg difference between the two sides. ° = degree

For an antegrade femoral nail, the distal locking screw(s) is removed, allowing the distal femur to rotate around the nail without shortening. The derotational maneuver is then performed as follows: the proximal femur derotational Schanz pin is used to stabilize the proximal femoral segment while the distal derotational pin is rotated in the desired direction; internally for a femoral retroversion deformity or externally for a femoral anteversion deformity. Once the appropriate amount of correction is obtained, the position of the derotational pins is held while a non-sterile operating room assistant utilizes the digital protractor application (Bonesetter Solutions, LLC, Ann Arbor, MI) to measure the obtained correction angle. Within the application, a photo is taken of the anterior reference pins from the foot of the bed and the software angle measurement tool is used. If inadequate correction is obtained, the derotational pins are adjusted accordingly and the new angle between the anterior reference is measured. This step is repeated until the desired correction is obtained (Figures 6A-6C). At this point, the correction is held by the assistant while a distal interlocking screw is placed utilizing the surgeon’s preferred technique or a technique specific to the implant. This will lock the rotation of the nail. It is recommended to lock the nail in the anterior-posterior holes or any unused lateral to medial holes (Figures 7A, 7B). We do not use the same holes as 15 to 30 degrees of rotation will not guarantee that an intact bony bridge will remain between drill holes after locking the nail a second time at the same location. A second locking screw is placed as well to secure the rotation because with a single locking screw there is 5 degrees of toggle possible and that can be problematic. All four Schanz pins are removed, and final fluoroscopic images are taken. A postoperative CT scanogram is obtained to confirm length, alignment, and degree of rotational correction (Figures 8A-8D, 9A-9D). For a retrograde nail the same procedure is followed placing a Schanz pin proximal to the nail to prevent shortening and the proximal locking screws removed.

## Results

From 2016 to 2021, 128 patients underwent femoral IM nailing for Winquist III and IV femur fractures. Of this cohort, 19 patients had a rotational malalignment greater than 15 degrees assessed by a post-operative CT Scanogram. Data from the 19 cases are outlined in Table 1. Age ranged from 18 to 55 with a mean age of 30.2. There were 9 antegrade and 10 retrograde nails. The cause of fracture was 13 gunshot wounds and 6 motor vehicle accidents. The fractures were defined by their location (proximal, midshaft, or distal) in addition to their AO and Winquist classification. The range of malrotation was 24 + 8 degrees (range: 18 to 47 degrees). These patients underwent an acute derotational procedure within three days of their index procedure. All patients were corrected to an average correction of 4.0 ± 2.1 degrees difference (range 0-8°) compared to the contralateral side. None of the patients required further surgeries to correct malrotation. There was no change in post-correction leg lengths.

**Table 1:** Data table of cases for femoral version correction GSW: gunshot wound, MVA: motor vehicle accident, AO OTA: Arbeitsgemeinschaft für Osteosynthesefragen (Association of the Study of Internal Fixation) Orthopaedic Trauma Association, I, II, III, IV: one, two, three, four

Case	Age	Nail	Cause	Location	AO OTA	Winquist	Difference in Version	After Correction
1	51	Antegrade	GSW	Midshaft	32-C	IV	19	4
2	22	Antegrade	MVA	Proximal shaft	32-C	IV	25.8	3
3	22	Antegrade	GSW	Distal third	32-C	IV	19.6	5
4	32	Antegrade	GSW	Midshaft	32-C	IV	17.6	6
5	25	Retrograde	GSW	Midshaft	32-C	IV	32.5	0
6	38	Retrograde	GSW	Midshaft	32-C	IV	37.7	1
7	49	Retrograde	GSW	Distal third	32-B	III	20.1	2
8	18	Retrograde	MVA	Midshaft	32-C	IV	47	5
9	41	Retrograde	MVA	Midshaft	32-B	III	19.7	3
10	19	Antegrade	GSW	Midshaft	32-C	IV	20.5	6
11	40	Antegrade	MVA	Proximal shaft	32-B	III	23.1	3.5
12	34	Retrograde	GSW	Distal third	32-B	III	18.7	6.7
13	24	Retrograde	GSW	Distal third	32-B	III	19.4	7.4
14	55	Retrograde	GSW	Midshaft	32-C	IV	28.2	8
15	18	Antegrade	MVA	Proximal shaft	32-C	IV	34	2
16	26	Antegrade	MVA	Midshaft	32-C	IV	27.3	3.5
17	23	Antegrade	GSW	Proximal shaft	32-C	IV	18.2	4
18	18	Retrograde	GSW	Distal third	32-C	IV	23.2	5
19	18	Retrograde	GSW	Distal third	32-C	IV	18.3	6

## Discussion

Malrotation of the femur after IM nailing is a difficult issue for orthopedic surgeons and is the most common complication following this procedure, with a prevalence of 19%-56% [1,5,7,10-14]. The normal anteversion in healthy individuals without deformity has been shown to be between 9 and 14 degrees [4,7]. The reported average side-to-side difference in anteversion between femurs is 4 degrees with a range of up to 13 degrees. Functional outcomes can be affected when the difference in the femoral version between the affected and contralateral femurs is greater than 15 degrees [3,5]. Malrotation greater than 15 degrees can lead to biomechanical changes in the coronal and sagittal plane which contribute to gait abnormalities from increased anterior pelvic tilt, changes in mean hip internal rotation, pelvic rotation, ankle range-of-motion, ankle peak plantar flexion, decreased peak hip and knee flexion, and knee range-of-motion [11,12]. Fractures that are comminuted, transverse, and outside of the diaphysis can increase the risk of malrotation [1]. Other risk factors for malrotation include patient positioning on a fracture table, unlocked nails, and repeat trauma after IM nailing. 

A variety of techniques have been developed to correct femoral rotational malalignment, but they all have their pitfalls and have not been shown to eliminate malrotation. Such techniques include aligning the cortical thickness of the fracture, using the relationship of the lesser trochanter and the patellar projection over the femoral condyles, and the relationship of the greater trochanter to the femoral head has been described [15,16]. These techniques have pitfalls that make them unreliable because of differences in fracture morphology and variation in normal anatomy [5,10]. The literature has described a technique that uses the inherent anteversion of second-generation antegrade femoral nails (11-12 degrees) to avoid malrotation [12,14]. This technique can reliably align the femur to the 11-12 degrees of anteversion which is built into the nail +/- 5 degrees. This technique does not consider the native version of the uninjured limb which may be outside of the 11-12 degrees of femoral anteversion. This is a technique used at our institution, yet we still had a malrotation incidence of 14.8%. The use of navigation for the assessment of the femoral version has been shown to have superior results compared to free-hand techniques. However, these navigation systems are expensive and increase the operative time. These techniques can be useful but have not shown the ability to eliminate malrotation. It is important to be aware of this complication and catch it early to offer a corrective procedure to prevent poor outcomes [19].

Measuring femoral anteversion on axial CT cuts also is not 100% reliable. The distal femoral measurement at the posterior condylar line is very reproducible but the femoral neck measurement has been noted to have a 5-degree variation depending on the cut used and measurer [17]. We used the best cut on both femurs and then had three individuals verify it. There are articles that have tried to standardize the measurement, but they also have variability in the proximal measurement [20].

At our institution, if there is a concern for a rotational malalignment during the postoperative evaluation, a CT scanogram is obtained which has been shown to be accurate in detecting malalignment [11]. Low-dose protocols can reduce radiation exposure by 90% and not affect the accuracy of the CT scanogram [13]. If a rotational difference of >15 degrees compared to the contralateral extremity is identified on a CT scanogram, surgery is offered, and our technique is utilized as soon as the patient is stable enough to return to the operating room. 

Nineteen of our patients with malrotation >15 degrees that was measured on CT scanogram were corrected acutely within three days of IM nailing. All patients were corrected to within 8 degrees of the non-injured side with a mean post-correction of 4.0 ± 2.1 degrees. There was no difference in pre-correction versus post-correction leg lengths measured on the CT scanogram. None of the 19 patients needed further corrective procedures and did well with physical therapy post-operatively. 

The goal of this technique was to provide an accurate, efficient, and reliable method as possible. This is the first use of a digital protractor used intraoperatively to aid in rotational correction. If a postoperative IM nail femur malrotation is identified early, preferably during the same hospital stay, this technique can save the patient from complications of malrotation. An early corrective procedure before bone healing has taken place can save the patient from the morbidity of revision IM nailing and corrective osteotomies. Using the strategic placement of Schanz pins, especially the most distal pin abutting the distal aspect of the nail, significantly minimizes loss of reduction, length, and alignment is minimized.

The major limitation of this technique is the inherent range of “normal” anteversion in individuals. The goal of this technique is to restore anteversion of the injured femur as close as we can to the uninjured limb. If the anteversion is within 15 degrees of the contralateral extremity, then the patient has been restored to their functional normal, even if it is not population normal. Secondly, although the Bonesetter application is a useful tool for measuring angles it is dependent on the user but can be minimized if images are taken correctly from the end of the bed from the correct angle.

## Conclusions

We present a technique for the correction of femoral malrotation utilizing simple orthopedic instruments, fluoroscopy, and a digital protractor through the Bonesetter application. This technique may be utilized in the acute, inflammatory phase of bone healing when the fracture is still mobile, typically in the first two weeks. Comminuted fractures have an incidence of malrotation after femoral nailing >15 degrees in 15% of the cases at our institution. This technique provides an efficient and accurate correction with the use of an intraoperative digital protractor, avoiding the need for revision IM nailing or osteotomies.
